# Efficacy of Save Medical Corporation (SMC)–01, a Smartphone App Designed to Support Type 2 Diabetes Self-Management Based on Established Guidelines: Randomized Controlled Trial

**DOI:** 10.2196/53740

**Published:** 2024-09-10

**Authors:** Nicholas Leung, Kayo Waki, Satoshi Nozoe, Shunpei Enomoto, Ryo Saito, Sakurako Hamagami, Toshimasa Yamauchi, Masaomi Nangaku, Kazuhiko Ohe, Yukiko Onishi

**Affiliations:** 1 Donald and Barbara Zucker School of Medicine Hempstead, NY United States; 2 Department of Planning, Information and Management University of Tokyo Hospital Tokyo Japan; 3 Department of Biomedical Informatics Graduate School of Medicine The University of Tokyo Tokyo Japan; 4 Department of Diabetes and Metabolic Diseases Graduate School of Medicine The University of Tokyo Tokyo Japan; 5 Save Medical Corporation Tokyo Japan; 6 Faculty of Biology University of Cambridge Cambridge United Kingdom; 7 Division of Nephrology and Endocrinology Graduate School of Medicine The University of Tokyo Tokyo Japan; 8 The Institute of Medical Science Asahi Life Foundation Tokyo Japan

**Keywords:** behavioral change, HbA_1c_, hemoglobin A_1c_, mHealth, randomized controlled trial, smartphone application, T2DM, diabetes mellitus, mobile apps, mobile app, type 2 diabetes, diabetes, self-management, Japan, multi-institutional, RCT, RCTs, efficacy, app development, safety, mobile phone

## Abstract

**Background:**

Lifestyle modifications are a key part of type 2 diabetes mellitus treatment. Many patients find long-term self-management difficult, and mobile apps could be a solution. In 2010, in the United States, a mobile app was approved as an official medical device. Similar apps have entered the Japanese market but are yet to be classified as medical devices.

**Objective:**

The objective of this study was to determine the efficacy of Save Medical Corporation (SMC)–01, a mobile app for the support of lifestyle modifications among Japanese patients with type 2 diabetes mellitus.

**Methods:**

This was a 24-week multi-institutional, prospective randomized controlled trial. The intervention group received SMC-01, an app with functions allowing patients to record data and receive personalized feedback to encourage a healthier lifestyle. The control group used paper journals for diabetes self-management. The primary outcome was the between-group difference in change in hemoglobin A_1c_ from baseline to week 12.

**Results:**

The change in hemoglobin A_1c_ from baseline to week 12 was –0.05% (95% CI –0.14% to 0.04%) in the intervention group and 0.06% (95% CI –0.04% to 0.15%) in the control group. The between-group difference in change was –0.11% (95% CI –0.24% to 0.03%; *P*=.11).

**Conclusions:**

There was no statistically significant change in glycemic control. The lack of change could be due to SMC-01 insufficiently inducing behavior change, absence of screening for patients who have high intention to change their lifestyle, low effective usage of SMC-01 due to design issues, or problems with the SMC-01 intervention. Future efforts should focus on these issues in the early phase of developing interventions.

**Trial Registration:**

Japan Registry of Clinical Trials jRCT2032200033; https://jrct.niph.go.jp/latest-detail/jRCT2032200033

## Introduction

In recent years, the number of patients with type 2 diabetes mellitus (T2DM) has been on the rise due to unhealthy diets, sedentary lifestyles, and an aging population [1]. The epidemic is only expected to worsen, with the number of adults with diabetes predicted to reach 415 million by 2040, costing the health care system US $802 billion a year [2]. Improving diabetes treatment is essential.

Treatment approaches for diabetes currently consist of pharmacological interventions and lifestyle modifications of diet and physical exercise. Lifestyle modifications have been shown to be effective in treating diabetes in several studies [3-5], enhancing insulin sensitivity and improving glycemic control [4]. Lifestyle interventions have improved diabetes control and cardiovascular risk factors and have reduced the use of diabetic medications [6]. They have shown a substantial and parallel reduction in glucose-lowering medication [7]. However, many patients struggle with adhering to lifestyle treatments [4,8]. This may be due to several factors, including socioeconomic and temporal factors that make it difficult to fully implement behavioral change [9]. There may be a lack of actionable advice from physicians and other health care workers on how to maintain a healthy lifestyle [10]. The way the advice is communicated is also important—a holistic understanding of the patient’s situation and motivational interviewing improve self-efficacy and adherence to lifestyle interventions [11-13].

Mobile health (mHealth; the use of mobile phones and other connected devices to improve health) could address some of these barriers. By using apps on mobile devices, mHealth enables remote monitoring of patients and delivery of personalized clinical advice [8]. mHealth has been shown to increase adherence to and efficacy of lifestyle interventions while reducing the financial burden on the health care system [14-17]. Mobile phone ownership rates reached 95% in developed regions in 2022 [18], and in Japan, the number of households owning smartphones reached 86.8% in 2020 [19]. Given this, and that nearly 0.5 billion people use health-related apps worldwide already, mHealth is likely to be a key addition to standard diabetes treatment [16].

Many diabetes-related health care apps have been introduced in Japan, but none are classified as medical devices, meaning they cannot be prescribed and can only be adopted by patients independently. In the United States, the app BlueStar (WellDoc Communications Inc) was approved by the Food and Drug Administration as a class II medical device in 2010 [20,21], becoming the first app in the world that could be prescribed by physicians. BlueStar provides personalized feedback in response to patient data per US diabetes treatment guidelines. Although the lifestyle of diabetes patients in Japan may differ from that of US patients, a similar app may be effective in combatting the disease in Japan, and trials in Japan have yielded promising outcomes [22,23]. The acceptance of mHealth among Japanese patients with lifestyle diseases is relatively high [24].

In this trial, we investigated the efficacy of Save Medical Corporation (SMC)–01, an app developed by Save Medical Inc designed to assist the self-management of T2DM by providing feedback according to the Japan Diabetes Society’s 2019 diabetes care guidelines and 2018-2019 diabetes treatment guide [25]. The main objective of our trial was to assess whether glycemic control could be improved through the usage of SMC-01 among patients with T2DM. We also studied whether any safety issues arose while using the app. We hypothesized that using the app would be beneficial to patients by helping them improve their glycemic control and lifestyle modification adherence.

## Methods

### Participant Inclusion Criteria and Recruitment

Patients were recruited from 4 medical institutions across Japan with significant experience in conducting clinical trials (Textbox 1). Recruitment was conducted by attending physicians during the patients’ regular consultations at outpatient clinics. Before patients enrolled in the study, the study’s supervising physician presented the review board–approved consent form to each patient and explained the contents, which included information about diabetes, how the app functions, how to operate the app, data collection, risks and benefits of participating, and compensation. Once the study was explained and it was determined that patients understood the explanation, patients were requested to participate in the study, and consent given by the patient’s own free will was collected in writing via the consent form.

**Participating institutions.**

- The Institute of Medical Science, Asahi Life Foundation
- Tokyo-eki Center Building Clinic
- Fukuwa Clinic
- Tokyo Center Clinic

Patients who met the inclusion criteria (Textbox 2) and did not meet the exclusion criteria (Textbox 3) were eligible for the study. To focus the study on patients most likely to benefit and most suitable for participation, the criteria selected patients who have inadequate glycemic control but have not recently changed their blood glucose management. Minors and in-patient participants were not eligible due to concerns of ethics and safety. Participants were also limited to those who own a smartphone. Patients who might expect changes in their blood glucose levels during the study, such as women who are already pregnant or who are planning on becoming pregnant, were not eligible. The study focused on the population with T2DM, excluding patients with type 1 diabetes mellitus due to differences in the pathophysiology between the 2 diseases.

Following obtaining consent and a subsequent 2-week observational period, patients were screened against the criteria, and eligible patients were enrolled in the study. The 24-week study began with a 12-week treatment period that included consultations every 4 weeks, followed by a 12-week treatment sustainment period that included 1 consultation. Patients were randomized into either the intervention group or the control group in a 1:1 ratio by block randomization (block size of 4) with baseline hemoglobin A_1c_ (HbA_1c_; less than 8% or 8% or greater) and hypoglycemic medication use (yes or no) as stratification factors. Patients were assigned an ID by the registration center for data tracking.

**Inclusion criteria of the study.**

- Outpatients who were aged 20 years or older when consent was obtained, regardless of sex.
- Patients who give written consent during visit 1.
- Patients who have been diagnosed with type 2 diabetes mellitus for at least 12 weeks (84 days) at visit 1.
- Patients who were currently using diet therapy or exercise therapy only, or who used diet therapy or exercise therapy in addition to diabetes medication and have had no change in their treatment for 12 weeks (84 days) or longer at the time of visit 1.
- Patients whose hemoglobin A_1c_ was 7% or greater and 9% or lower at the time of visit 1.
- Patients who had a smartphone and had been using it for 12 weeks (84 days) or longer at the time of visit 1.
- Patients who could enter information into the device being studied without difficulty as determined by the study’s supervising physician.

**Exclusion criteria of the study.**

- Patients who were planning on conceiving, were currently pregnant, or who were nursing.
- Patients who are premenopausal and who tested positive on a urine pregnancy test.
- Patients who had been diagnosed with type 1 diabetes mellitus.
- Patients who had been diagnosed with secondary diabetes mellitus.
- Patients who received an insulin injection within 12 weeks (84 days) of visit 1.
- Patients who required the assistance of a 3rd party in treatment of hypoglycemia in the past.
- Patients who had been diagnosed with and were currently undergoing treatment for proliferative diabetic retinopathy.
- Patients who had developed cardiovascular disease within 12 weeks (84 days) of visit 1.
- Patients with decompensated heart failure.
- Patients with severe liver impairment (alanine transaminase, as measured at the central testing location during visit 1) greater than 3 times the standard maximum value.
- Patients with kidney disease (estimated glomerular filtration rate less than 45 mL/min/1.73m^2^ or urine albumin of 300 mg/gCre or higher, both as measured at the central testing location during visit 1).
- Patients with a chronic illness that required treatment with continuous pharmacotherapy (oral, injected, or inhaled) such as corticosteroids, immunosuppressants, or loop diuretics.
- Patients with malignancy (less than 5 years without recurrence) or a communicable disease (sepsis).
- Patients who planned to be admitted to a hospital or undergo surgery during the study period.
- Patients with drug addiction, alcohol use disorder, or an unstable psychological illness.
- Patients who were limited in their physical activity due to a medical condition other than diabetes.
- Patients whose hemoglobin A_1c_ value differed by more than 1% between visit 1 (measured at the hospital) and when measured at some point 4 to 10 weeks before visit 1.
- Patients who planned to, were currently, or had (within the past 12 weeks; 84 days) been enrolled in a different clinical trial for a pharmacologic agent or medical device, or a postmarketing clinical trial.
- Patients who, within the past 12 weeks (84 days), had used a mobile app on their smartphone or have been self-monitoring their blood glucose for diabetes self-management, and whose evaluation of the efficacy of the device in this study might be influenced by said experience, as determined by the study’s supervising physician.
- Patients who plan to switch their smartphone within 24 weeks of visit 2.
- Medical staff involved in this experiment and their close relatives.
- Save Medical employees or employees of companies that had been hired to complete tasks related to this experiment.
- Patients who could not comply with or refused to comply with conditions put forth by study staff, such as rules for visiting the hospital, or rules for taking medication.
- Patients determined by this study’s supervising physician to be unfit for involvement in this study for any other reason.

### Study Design

This study was a multi-institutional, prospective, and unblinded 2-armed randomized controlled trial carried out across 4 medical institutions in Tokyo, Japan. We chose to conduct an unblinded trial because we predicted that the use of a placebo app with minimal function would lead to the control group unblinding themselves.

We provided both groups with Bluetooth-enabled blood pressure cuffs (A&D, UC-352BLE) and scales (A&D, UA-651BLE). Patients in the intervention group were instructed to install the SMC-01 app onto their smartphones and use it to track diet, medication adherence, exercise, body weight, and blood pressure. Recordings of blood pressure and weight were collected automatically through the Bluetooth-enabled devices but could also be input manually. The rest of the data was input manually.

**Figure 1:**
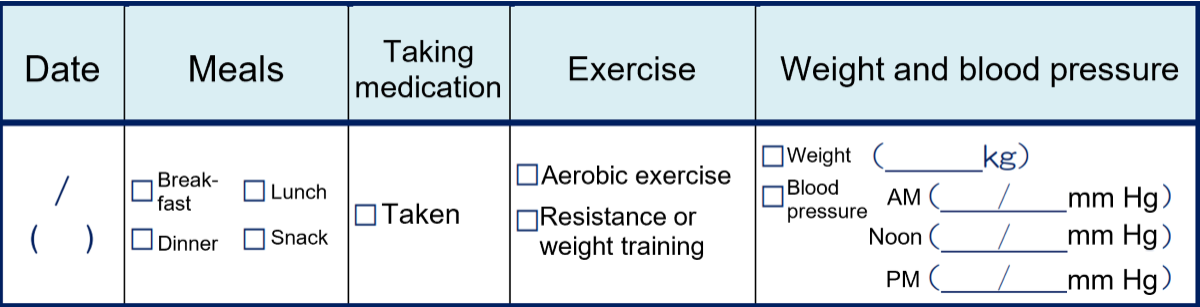
Entry in patient journal (English translation).

Patients in the control group recorded information about their diabetes self-management in a journal. They were asked to record which meals they ate (breakfast, lunch, dinner, or snack), whether they took their diabetes medication, whether they exercised and what type of exercise (aerobic or resistance training), their weight in kilograms, and their blood pressure along with when they measured it (morning, noon, or evening). Unlike the SMC-01 group, the control group did not record the content of meals, and only recorded whether they ate a meal or not. The format of a journal entry is shown in Figure 1.

### SMC-01 Design

**Figure 2:**
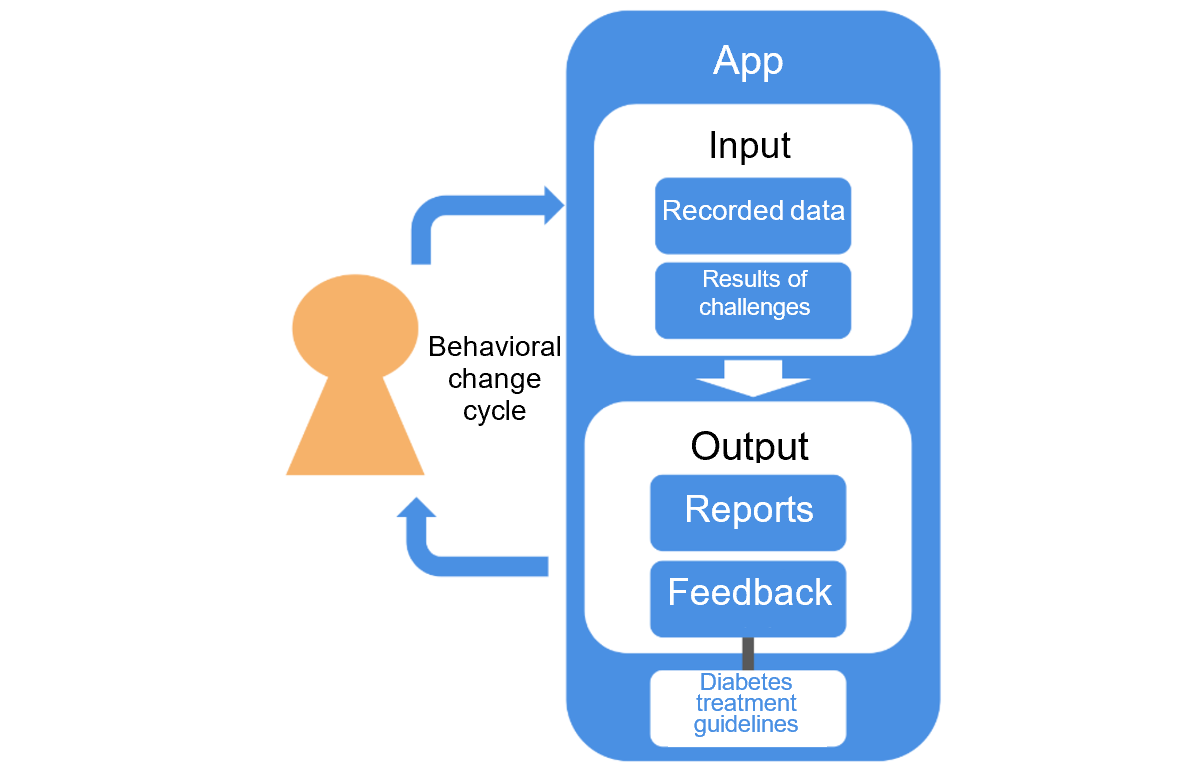
Overview of SMC-01. SMC: Save Medical Corporation.

SMC-01 (Figure 2) was designed to assist the treatment and management of T2DM, complementing, not replacing, physicians. SMC-01 breaks down goals set by physicians into smaller and specific actions and sends them to patients as daily feedback and challenges. Through this, it aims to increase the likelihood of long-term behavioral change by making goals more achievable and by addressing the issue of patients not knowing how to implement their physician’s advice.

SMC-01 consists of 4 main functions: facilitating data input, providing feedback in response to the data input, sending reminders, and setting daily challenges.

**Figure 3:**
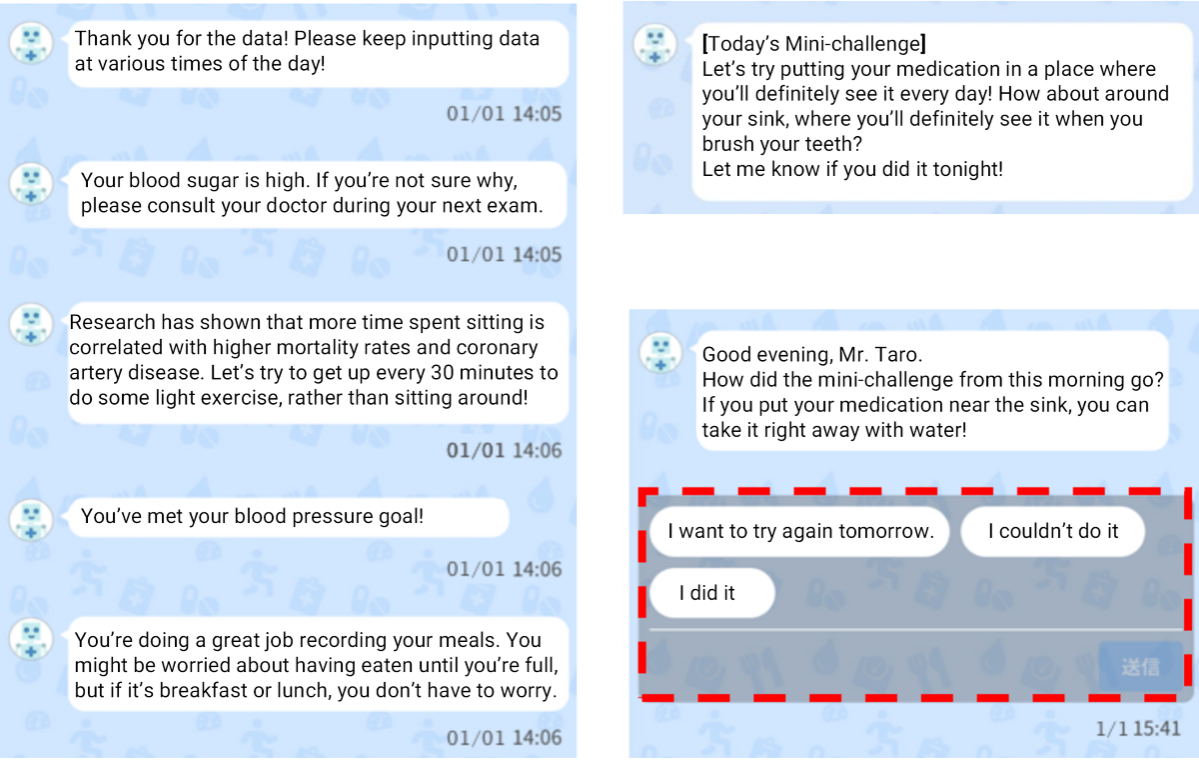
Example messages and challenges sent by Sketto (English translation).

Patients receive feedback per Japanese diabetes treatment guidelines based on their daily behavior. This is implemented using Sketto, a chatbot within SMC-01 that sends messages to the patient based on patient-entered data. The content of these messages depends largely on whether the data the patient enters is within normal limits according to Japanese diabetes treatment guidelines. If the patient’s data are within normal limits, Sketto praises the patient for their hard work and encourages them to continue their diabetes self-management as well as their data logging. On the other hand, if the patient’s data are outside of normal limits, Sketto warns the patient about the possible implications of the measurement, such as poor health outcomes, and encourages the patient to seek the advice of a health professional. For example, when the patient records their weight, Sketto sends a message providing positive feedback on healthy lifestyle choices or caution on unhealthy lifestyle choices depending on the patient’s BMI, which SMC-01 calculates from the patient’s height registered at the initial set-up. When the patient records his or her meal data, Sketto sends a message depending on the patient’s reported satiety level and the time of day. For medication adherence, Sketto sends reminders if patients forget to record that they took their medication, and praises patients for recording medication adherence several days in a row. Examples of some of these messages are shown in Figure 3.

Sketto also sends patients health-related articles to increase patients’ knowledge about diabetes and health in general. Unlike the feedback described above, these articles are not personalized based on patient data. Patients can also review their records in the form of reports. These reports show the patient’s self-management over time, such as how many days the patient has exercised, their change in weight, and the days they took their medication. The supervising physicians of the study could review the patients’ self-management by viewing the app’s screen. Review of data input into the app by the study’s supervising physicians could be accomplished at any time, but the review was mainly carried out during visits 3 through 6.

Every morning, Sketto sends patients mini-challenges within the app that are designed to augment the patients’ assigned diet, medication adherence, and exercise-based behavior modifications. These challenges begin at the lowest level, and, depending on whether the patient succeeds, a similar challenge that is slightly harder or easier may be assigned the following day. Otherwise, a mini-challenge from another category will be assigned. The patient can also choose to repeat the same challenge on the following day. Examples of mini-challenges can be found in Table S1 in Multimedia Appendix 1.

### Data Collection

**Figure 4:**
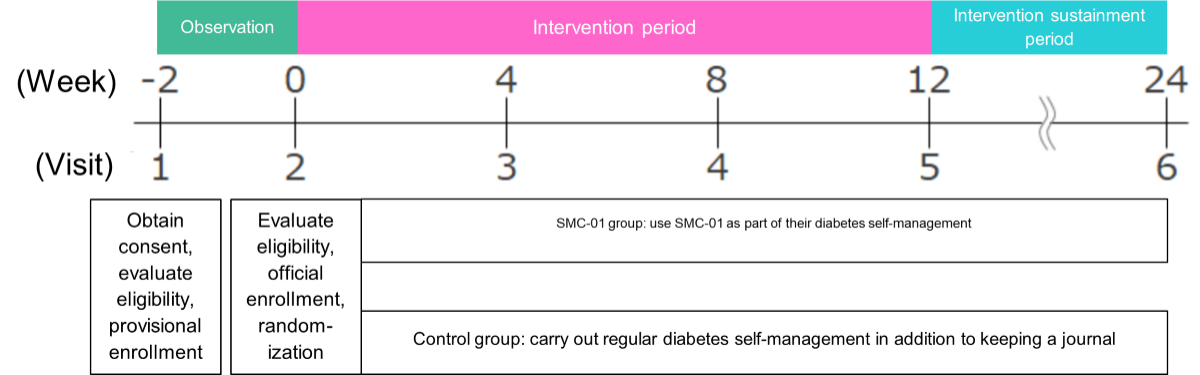
Overview of the study timeline. SMC: Save Medical Corporation.

Initial measurements were made at visit 1 at the beginning of the observation period. The study (Figure 4) included 6 in-person visits, a 2-week observation period, a 12-week intervention period, and a 12-week intervention sustainment period. If patients withdrew from the study at any point, observation and testing were carried out when the patient came to the health care facility to withdraw from the study. A detailed timeline of the study can be found in Table S2 in Multimedia Appendix 1.

From visit 1 until visit 6 or until measurement of efficacy outcomes had been completed, physicians in the study were prohibited from providing specific calorie intake and exercise amount goals to patients. Nonspecific advice, such as general directions to eat more slowly or eat less salt, was permitted. These limitations were set to ensure that changes in exercise amount and calorie intake could be attributed to the app and not instructions from physicians.

### Outcome Measures

The primary outcome was the between-arm difference in change in HbA_1c_ values between the baseline and the end of the intervention period.

The study evaluated the following secondary measurements:

- HbA_1c_ value: between-arm difference in the change from baseline to the end of the intervention sustainment period
- Fasting blood glucose: between-arm difference in the change from baseline to the end of the intervention period and to the end of the intervention sustainment period.
- Fasting insulin: between-arm difference in the change from baseline to the end of the intervention period and to the end of the intervention sustainment period.
- Fasting intact-proinsulin or insulin ratio: between-arm difference in the change from baseline to the end of the intervention period and to the end of the intervention sustainment period.
- Self-maintenance adherence rate (diet, exercise, and medication adherence; limited to patients taking diabetes medication)

Self-maintenance adherence rates were calculated as follows:



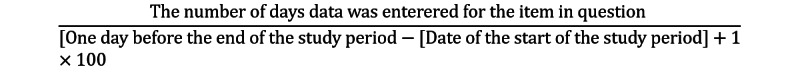



### Ethical Considerations

The study was registered in the Japan Registry of Clinical Trials (jRCT2032200033) on May 17, 2020. It was approved by the research ethics committee of The Institute for Adult Diseases, Asahi Life Foundation (179-8). We conducted the study per the Ministerial Ordinance on Standards for Conducting Clinical Trials of Medical Devices (The Ministerial Ordinance on Good Clinical Practice). The study has been reported as per the eHealth CONSORT (Consolidated Standards of Reporting Trials) statement. We obtained written informed consent from all participants before the study, and study data have been anonymized. Participants were compensated JP ¥10,000 (roughly US $64) for their time.

### Statistical Analysis

We set the mean difference in the change in HbA_1c_ from the baseline that we were designing the study to detect to 0.5, as this is generally considered a clinically significant change in HbA_1c_ [26,27]. The SD common to both groups was set to 1 based on values from previous studies [23,28,29], and the 2-sided significance level was 5% with 90% power. The resulting required sample size is 86 for each group. Taking into account the likelihood of dropouts, the target sample size for each group was set at 100 cases (200 cases in total).

Per the intention-to-treat (ITT) analysis principle, all randomly assigned patients were included in the full analysis set (FAS). In the case that a patient was excluded from the analysis, justification for the exclusion was documented. Patients in the FAS who met all of the following criteria that did not violate the selection exclusion criteria and protocol were included in the per protocol set (PPS):

- Met the inclusion criteria and did not meet the exclusion criteria
- HbA_1c_ value both at baseline and at the end of the intervention period was not missing
- No use of any of the prohibited medications, used conditional medications according to the study guidelines, and did not carry out any other prohibited treatments
- Self-management adherence rate above 80%
- Did not commit any serious study protocol violations by the end of the intervention period

All patients that participated in the intervention period of the study were included in the safety analysis set. Demographic data and baseline values were compared between groups using the Fisher exact test for discrete values and a 2-tailed *t* test for continuous values.

As the primary analysis, an analysis of covariance was performed on the change in HbA_1c_ from baseline to the end of the intervention period (wk 12) in the FAS, with baseline values as the covariate, and the between-group differences and their 95% CIs were calculated. As a sensitivity analysis, the same analysis was performed for the PPS and for patients with HbA_1c_ measured at all time points (wk 0, 4, 8, 12, and 24). In addition, the FAS was analyzed with a linear mixed effects model that included all time points. The objective variable was the change in HbA_1c_ from baseline, the random effect was the patient, and the fixed effects were group, time point, group × time point interaction, and baseline HbA_1c_. The amount of change for each group, the between-group difference in change, and their 95% CIs at each time point were calculated.

As a secondary analysis, an analysis of covariance was performed in the FAS on the change from baseline in HbA_1c_, fasting blood glucose, fasting insulin, and fasting intact-proinsulin or insulin ratio at the end of the intervention period (wk 12) and at the end of the intervention sustainment period (wk 24) with baseline values as covariates. In addition, summary statistics of change and measurements were calculated for each time point (wk 0, 4, 8, 12, and 24) for each group. The percentage of HbA_1c_ less than 7% at each time point (wk 4, 8, 12, and 24) for each group was also calculated. However, the denominator was the FAS population excluding patients with HbA_1c_ less than 7% at baseline. Self-maintenance adherence rates for diet, exercise, and medication were assessed in the following time periods and compared between groups by a 2-tailed *t* test: week 0 to week 4, week 4 to week 8, week 8 to week 12, week 12 to week 24, week 0 to week 12, week 0 to week 24.

In addition, the same analyses on HbA_1c_ levels were performed for the following subgroups as in the primary and secondary analyses.

- Sex: female or male
- Age: younger than 65 years or 65 years or older
- Baseline HbA_1c_: less than 8% or 8% or greater
- Baseline BMI: less than 25 kg/m^2^ or 25 kg/m^2^ or greater
- Hypoglycemic medication usage at week 2: yes or no
- Self-management adherence rate during the intervention period: less than 80% or 80% or greater

For analysis of safety for the safety analysis set, adverse events were classified for each group using system organ classes and preferred terms of the Japanese version of the Medical Dictionary for Regulatory Activities (version 23.0) [18], and the number of patients and occurrence rate for each system organ class or preferred term categories were tabulated. Additionally, the occurrence rates were compared between groups by Fisher exact test. For bugs in the SMC-01 group, the number of patients with bugs, occurrence rate, and the number of cases were tabulated. For each clinical test item and vital sign, levels and the difference from the beginning of the intervention period were calculated at each time point in each group as summary statistics.

All statistical tests were 2-tailed, with a significance level of 5%. CIs were calculated as 2-tailed 95% CIs. SAS (version 9.4; SAS Institute Inc) was used for statistical analysis.

## Results

### Overview

**Figure 5:**
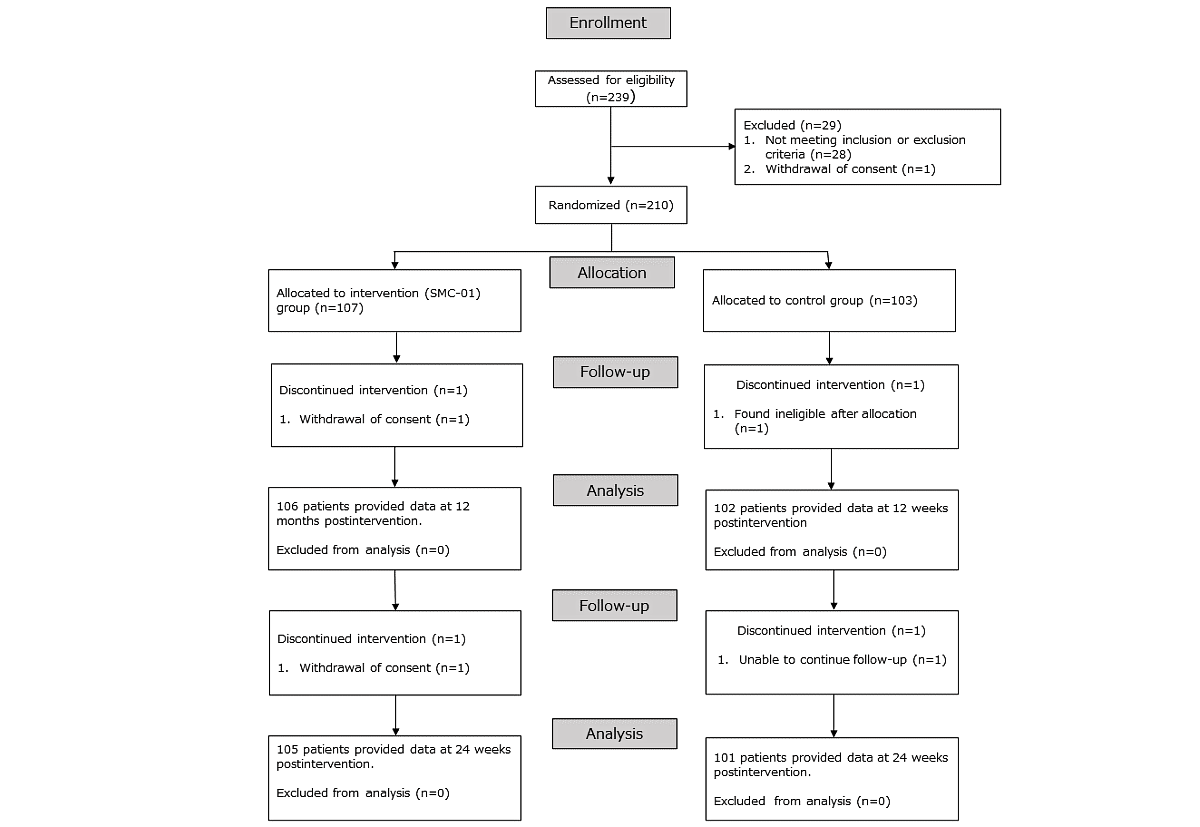
CONSORT flow diagram. CONSORT: Consolidated Standards of Reporting Trials; SMC: Save Medical Corporation.

This study was conducted from May 11, 2020, to April 5, 2021. In total, 210 participants were randomly assigned between groups, yielding 107 (intervention) and 103 (control) at baseline, 106 (intervention) and 102 (control) at the end of 12 weeks, and 105 (intervention) and 101 (control) at the end of 24 weeks (Figure 5). Patients were well matched in baseline characteristics (Table 1), although urine albumin or creatinine ratio was significantly higher in the intervention group than in the control group (42.50 mg/g of Cr in the intervention group versus 20.82 mg/g of Cr in the control group, *P*=.01).

**Table 1:** Baseline characteristics^a^.

Characteristics		SMC^b^-01 group (n=107)	Control group (n=103)	*P* value^c^	
**Age (years), mean (SD)**		57.6 (8.35)	58 (8.79)	.75	
	<65, n (%)	83 (77.6)	78 (75.7)	.87	
	≥65, n (%)	24 (22.4)	25 (24.3)		
**Sex, n (%)**					.60
	Female	22 (20.6)	18 (17.5)		
	Male	85 (79.4)	85 (82.5)		
Disease duration (years), mean (SD)		10.93 (5.908)	10.10 (5.334)	.29	
Height (cm), mean (SD)		167.04 (8.207)	167.62 (6.915)	.58	
Weight (kg), mean (SD)		73.80 (14.668)	73.81 (14.163)	.99	
**BMI (kg/m^2^), mean (SD)**		26.34 (4.283)	26.18 (4.197)	.78	
	<25, n (%)	45 (42.1)	47 (45.6)	.68	
	≥25, n (%)	62 (57.9)	56 (54.4)		
Waist circumference (cm), mean (SD)		92.61 (11.255)	92.16 (10.207)	.77	
**Insulin dependence or independence, n (%)**					
	Insulin dependent	0 (0)	0 (0)		
	Insulin independent	107 (100)	103 (100)		
C-peptide (ng/mL), mean (SD)		2 (0.799)	1.97 (0.829)	.78	
Urine albumin or creatinine ratio (mg/g of Cr), mean (SD)		42.50 (84.277)	20.82 (31.552)	.01	
**Baseline HbA_1c_, mean (SD)**		7.74 (0.554)	7.67 (0.542)	.37	
	<8, n (%)	71 (66.4)	74 (71.8)	.46	
	≥8, n (%)	36 (33.6)	29 (28.2)		
**Hypoglycemic medication use, n (%)**					.72
	Do not use	20 (18.7)	17 (16.5)		
	Use	87 (81.3)	86 (83.5)		

^a^Baseline values were taken at the beginning of the intervention period (visit 2). If there was no measurement from the beginning of the intervention period, the latest value before the start of the intervention period was used.

^b^SMC: Save Medical Corporation.

^c^*P* value: Discrete variables were analyzed using the Fisher exact test. Continuous variables were analyzed using an unpaired 2-tailed *t* test.

### Self-Management Adherence Rates

Overall adherence rates were high during the intervention period (Table 2), at 82.28% in the SMC-01 group and 79.35% in the control group (*P*=.20). Average diet adherence rates were significantly higher in the control group than in the SMC-01 group at 4 weeks (96.39%, SD 10.67% and 99.01%, SD 2.42%, respectively; *P*=.02) and week 24 (91.57%, SD 22.44% and 97.63%, SD 10.37%, respectively; *P*=.01). Medication adherence rates were similar in both groups, with no statistically significant differences found at any time point (all *P*>.05). Average exercise self-management adherence rates were significantly higher in the SMC-01 group than in the control group, with 60.79% (SD 37.22%) and 49.23% (SD 37.92%; *P*=.03) at 4 weeks, 60.32% (37.24%) and 49.33% (38.52%; *P*=.04) at 8 weeks, and 59.29% (35.95%) and 47.34% (37.01%; *P*=.03) at the end of the intervention period.

**Table 2:** Self-management adherence rate^a^.

Adherence rate and period		SMC^b^-01 group	Control group	*P* value^c^
**Overall**				
	Wk 0-12, mean (SD)	82.28 (16.56)	79.35 (16.27)	.20
	≤80%, n (%)	40 (37.4)	50 (48.5)	.13
	>80%, n (%)	67 (62.6)	53 (51.5)	
**Meal, mean (SD)**				
	Wk 0-4	96.39 (10.67)	99.01 (2.42)	.02
	Wk 4-8	96.63 (12.28)	98.87 (2.63)	.07
	Wk 8-12	95.67 (12.96)	97.94 (10.03)	.16
	Wk 12-24	91.57 (22.44)	97.63 (10.37)	.01
	Wk 0-12	95.75 (11.18)	97.79 (9.70)	.16
	Wk 0-24	93.16 (14.98)	97.27 (9.78)	.02
**Medication, mean (SD)**				
	Wk 0-4	94.15 (15.23)	96.90 (11.05)	.18
	Wk 4-8	95.22 (14.38)	96.43 (11.44)	.55
	Wk 8-12	94.59 (12.35)	95.57 (13.65)	.62
	Wk 12-24	94.13 (16.48)	96.27 (11.97)	.33
	Wk 0-12	94.05 (14.67)	95.46 (14.30)	.52
	Wk 0-24	93.68 (14.69)	95.53 (12.69)	.38
**Exercise, mean (SD)**				
	Wk 0-4	60.79 (37.22)	49.23 (37.92)	.03
	Wk 4-8	60.32 (37.24)	49.33 (38.52)	.04
	Wk 8-12	57.88 (37.49)	47.97 (37.45)	.06
	Wk 12-24	55.27 (38.55)	46.02 (38.87)	.09
	Wk 0-12	59.29 (35.95)	48.34 (36.89)	.03
	Wk 0-24	57.05 (35.80)	47.34 (37.01)	.06

^a^The overall self-management adherence rate was defined as the average of diet, exercise, and medication (only for those taking medication) adherence rates.

^b^SMC: Save Medical Corporation.

^c^*P* value: Discrete variables were analyzed using the Fisher exact test. Continuous variables were analyzed using an unpaired 2-tailed *t* test.

### Efficacy of SMC-01

Based on the analysis of covariance (Table 3), the change in HbA_1c_ from baseline to the end of the intervention period (12 wk) was –0.05% (95% CI –0.14% to 0.04%) in the SMC-01 group and 0.06% (95% CI –0.04% to 0.15%) in the control group, with a between-group difference (SMC-01 group – control group) of –0.11% (95% CI –0.24% to 0.03%, *P*=.11). The difference is not statistically significant.

Secondary analysis of the change in HbA_1c_ from baseline to the end of the intervention sustainment period (24 wk) revealed a between-group difference (SMC-01 group – control group) of –0.16% (95% CI –0.36% to 0.03%; *P*=.11). The difference is not statistically significant.

Secondary analysis of the change in fasting blood glucose from baseline to the end of the intervention period (12 wk) yielded a between-group difference (SMC-01 group – control group) of –4 (95% CI –10.6 to 2.6; *P*=.23). From baseline to the end of the intervention sustainment period (24 wk) the between-group difference (SMC-01 group – control group) was –4.8 (95% CI –12.6 to 3; *P*=.23). The differences are not statistically significant.

Secondary analysis of the change in fasting insulin from baseline to the end of the intervention period (12 wk) yielded a between-group difference (SMC-01 group – control group) of 0.24 (95% CI –0.64 to 1.13; *P*=.59). From baseline to the end of the intervention sustainment period (24 wk) the between-group difference (SMC-01 group – control group) was 0.19 (95% CI –0.47 to 0.85; *P*=.56). The differences are not statistically significant.

Secondary analysis of the change in fasting intact-proinsulin or insulin ratio from baseline to the end of the intervention period (12 wk) yielded a between-group difference (SMC-01 group – control group) of –0.01 (95% CI –0.04 to 0.02; *P*=.44). From baseline to the end of the intervention sustainment period (24 wk) the between-group difference (SMC-01 group – control group) was –0.01 (95% CI –0.03 to 0.01; *P*=.42). The differences are not statistically significant.

In the sensitivity analysis of the PPS and the complete set, ANCOVA of the between-group difference in the average change in HbA_1c_ from baseline to the end of the intervention period (wk 12) was not statistically significant (*P*=.14 and *P*=.11). Mixed model estimates of the change in HbA_1c_ from baseline in the FAS yielded a statistically significant difference at week 8 of –0.12 points (*P*=.04) and week 12 of –0.12 points (*P*=.04). The full sensitivity analysis results have been included in Multimedia Appendix 1.

In subgroup analysis of change in HbA_1c_ from baseline to the end of the intervention period (wk 12), statistically significant differences were found in the aged younger than 65 years subgroup (estimated values in the SMC-01 group and control group were –0.07% and 0.08%, respectively, *P*=.04, same order in subsequent listings) and the baseline HbA_1c_ less than 8% subgroup (–0.04% and 0.11%; *P*=.04). Subgroup analysis of change in HbA_1c_ from baseline to the end of the intervention sustainment period (wk 24) revealed no statistically significant difference between the 2 groups at any time point. The full subgroup analysis results have been included in Multimedia Appendix 1.

**Table 3:** Summary of efficacy analyses^a,b,c,d^.

		SMC^e^-01 group (n=107)^f^	Control group (n=103)^e^	Difference^e^	*P* value
**HbA_1c_^g^ (% points)**					
	Wk 12	–0.05 (–0.14 to 0.04)	0.06 (–0.04 to 0.15)	–0.11 (–0.24 to 0.03)	.11
	Wk 24	0.11 (–0.03 to 0.25)	0.28 (0.13 to 0.42)	–0.16 (–0.36 to 0.03)	.11
**Fasting blood glucose (mg/dL)**					
	Wk 12	–3.2 (–7.8 to 1.4)	0.9 (–3.9 to 5.6)	–4.0 (–10.6 to 2.6)	.23
	Wk 24	1.7 (–3.8 to 7.1)	6.5 (0.9 to 12.0)	–4.8 (–12.6 to 3.0)	.23
**Fasting insulin (μU/mL)**					
	Wk 12	0.17 (–0.45 to 0.79)	–0.08 (–0.71 to 0.56)	0.24 (–0.64 to 1.13)	.59
	Wk 24	–0.11 (–0.57 to 0.35)	–0.30 (–0.78 to 0.17)	0.19 (–0.47 to 0.85)	.56
**Fasting intact-proinsulin or insulin ratio**					
	Wk 12	–0.01 (–0.03 to 0.01)	0.00 (–0.02 to 0.02)	–0.01 (–0.04 to 0.02)	.44
	Wk 24	–0.02 (–0.03 to 0.00)	–0.01 (–0.02 to 0.01)	–0.01 (–0.03 to 0.01)	.42

^a^The response variable is the absolute change from baseline, with the baseline value as the covariate in the analysis of covariance.

^b^Baseline measurement was measured at the start of the intervention period (visit 2). If there was no measurement at the start of the intervention period, the last measurement before the start of the intervention period was used.

^c^If there was no measurement at the 12-week visit, the last measurement taken between the second day and the 92nd day of the study was used.

^d^If there was no measurement at the 24-week visit, the last measurement taken between the second day and the 175th day of the study was used.

^e^SMC: Save Medical Corporation.

^f^Data are expressed as mean absolute change from baseline and its 95% CI.

^g^HbA_1c_: hemoglobin A_1c_.

### Safety of SMC-01

The results of the safety analysis are included in Multimedia Appendix 1. There were no adverse events or deaths caused by the device under study.

## Discussion

### Principal Findings

We did not find any statistically significant difference in outcomes between the SMC-01 and control groups. Further, HbA_1c_ values essentially did not change in either group. Patients who participate in clinical trials normally experience a change in health outcomes due to the Hawthorne effect and the placebo effect, regardless of the intervention. These effects in this study may have been small because this trial was conducted during the COVID-19 pandemic, when people were advised to stay home, possibly reducing changes in behavior such as exercise.

We can assess possible reasons for the failure of the intervention to change outcomes using behavioral change theory, specifically, the Theory of Planned Behavior [30]. When designing a lifestyle intervention, it is critical to view it as requiring a behavioral change, a perspective that was not used in the SMC-01 intervention. The first step is to clearly define the desired change in behavior. In this study, the desired change was a loose collection of behaviors associated with following physician guidance on a healthy lifestyle. This may have been too vague to be actionable compared to past successful interventions in diet and exercise that defined concrete actions [31-33]. The next consideration is the baseline lifestyle of the patients. It is possible that patients were already following physician lifestyle instructions, and that there was not much need for further lifestyle intervention via SMC-01.

Intention is key in the Theory of Planned Behavior framework. The intervention sought to have patients make a behavior change large enough to produce a significant change in glycemic control. Behavioral change is difficult for most people and requires strong motivation, and motivation was not evaluated as one of the inclusion criteria of this trial. If a patient does not intend to change or perform a behavior, they are very unlikely to do so [30]. Other studies have had success screening for patients who are in the contemplation, preparation, or action stages of the transtheoretical model of behavioral change, increasing the likelihood of strong motivation [34-36]. A 2015 study showed that 92% of Japanese patients with T2DM met this criterion [37]. A different intervention would be appropriate for patients who are not motivated to change [38]. Our study did not specify any screening test for high intention.

Another concern is the lack of clear information about patient behavior. The study examined elements of patient behavior such as app usage but was highly focused on the ultimate health changes rather than behavior. We can assume that the desired behavioral change would have led to improved glycemic control, as has been shown in many other studies [31,32,39], but we do not have enough data to confirm whether their behavior truly changed. Future studies of lifestyle intervention should carefully investigate how to measure behavioral changes. Additionally, SMC-01 may not have been used as intended due to problems with the protocol and its implementation. There may have been insufficient intervention design features. The study found high levels of usage, and yet the intervention did not change glycemic control. In this study, usage was measured as the percentage of days for which data was input at least once. User engagement with other features was not measured. These usage measurements may not have been the appropriate indicators of effective usage of SMC-01. Studies have shown that simply recording data does not improve health care outcomes [40,41]. A review of 32 studies found that adherence rates in mHealth interventions were inconsistent [42]. Screen-time measurements may be used to determine how often patients accessed certain features, providing insight into patient engagement with feedback and whether the patient truly received the intervention.

Another concern is possible insufficient data entry. Objective health data are crucial to mHealth interventions, as self-reported data are often unreliable [43,44]. Automatic data collection objectively measures behavior and was used for the measurement of weight and blood pressure in this study. These are intermediate health changes on the way to changed glycemic control, not behaviors. Future studies should apply automatic data collection of behavioral change. Finally, there may have been insufficient behavioral change information and directions suggested by SMC-01. Closed feedback loops form the backbone of mHealth interventions, but this feedback must be personalized and actionable [45-47].

We do not have sufficient data from this study to determine which, if any, of these possible issues contributed to the outcome. In future studies, addressing these possible issues during the study and app design phase is recommended. In future app design, a theoretical framework of behavioral change can be used to guide the implementation of features. It has been shown that basing interventions on behavior change theory improves health outcomes [47].

This study has some limitations. It was conducted in a Japanese population, and there are known differences between Japanese and other populations about lifestyle and the pathophysiology of T2DM [48,49]. This study was limited to patients who owned smartphones, which may have led to biases related to digital literacy and socioeconomic status. Social desirability bias also likely influenced results due to a lack of blinding.

To our knowledge, this study was the first trial in Japan that sought to obtain official approval for a smartphone app for T2DM as a medical device and will serve as a foundation for the design of future apps and clinical trials seeking medical device registration. mHealth devices for the management of chronic diseases will continue to improve, and future studies in Japan should continue to focus on gaining official approval. Information from this trial, along with the discussion of possible shortfalls, should help improve future efforts.

### Conclusions

No statistically significant between-arm difference was found in the change in HbA_1c_ from baseline to the end of the 12-week intervention period. Future studies should improve clarity regarding the theory of the intervention, improve the methods used, and ensure the measurement of behavior and use throughout the study.

## Appendix

Multimedia Appendix 1 Tables depicting examples of mini-challenges, study schedule, summary of sensitivity analyses for HbA_1c_, summary of subgroup analyses for HbA_1c_, all adverse events, and breakdown of bugs and sections depicting PPS definition and safety analysis. Hb: hemoglobin; PPS: per protocol set.

Multimedia Appendix 2 CONSORT-eHEALTH checklist (V 1.6.1).

## Abbreviations

FAS: full analysis set
HbA_1c_: hemoglobin A_1c_
ITT: intention-to-treat analysis
mHealth: mobile health
PPS: per protocol set
SMC: Save Medical Corporation
T2DM: type 2 diabetes mellitus
