# Supplementary material for: Efficacy of Save Medical Corporation (SMC)–01, a Smartphone App Designed to Support Type 2 Diabetes Self-Management Based on Established Guidelines: Randomized Controlled Trial
Source: J Med Internet Res. 2024 Sep 10;26:e53740. doi: 10.2196/53740 (PMC11429663; doi:10.2196/53740)
Supplement: Multimedia Appendix 1 [file jmir_v26i1e53740_app1.docx]

**Multimedia Appendix 1.** Tables depicting examples of mini-challenges, study schedule, summary of sensitivity analyses for HbA1c, summary of subgroup analyses for HbA1c, all adverse events, and breakdown of bugs and sections depicting PPS definition and safety analysis. Hb: hemoglobin; PPS: per protocol set.

**Table S1.** Examples of mini-challenges.

| Exercise mini-challenges in order of increasing difficulty included:   1. When told to “exercise,” do you feel like saying “that’s impossible!” and give up from the beginning? As a matter of fact, there are easy, effective exercises that you can do at home. For example, wiping up with a cloth instead of using a vacuum cleaner, or using a broom to sweep places that a vacuum cleaner can’t reach are easy and effective ways to exercise. Let’s try incorporating some exercise into our everyday life. 2. How about trying to do 30 calf raises while washing the dishes or handing the laundry? 💪 You can even do it while waiting at the cash register or the crosswalk. Let’s try doing some calf raises whenever we’re standing! 3. The timing of exercise is very important. Blood sugar levels rise the most 30-60 minutes after a meal. Exercising during that time is an effective way to curb that rise in blood sugar. Try being conscious of doing an appropriate amount of exercise or chores after eating. |
| --- |
| Diet mini-challenges in order of increasing difficulty included:   1. What do you usually drink? When drinking your favorite beverages, make sure to check the calorie count and nutritional information on the label! It’s common to hear people say that drinks had more calories than they thought 😞, especially for clear drinks. Definitely try it out today. I’ll ask you how it went tonight. 2. It’s easy to drink a lot, especially when you’re thirsty. Try drinking water or unsweetened tea first to lower the amount of sugar you get from drinks 😛 Remember, drinks like milk tea already have sugar in them, so be careful! 3. Have you seen the amount of sugar in soft drinks before? Sports drinks have 25-35 g per bottle, and even a 150 ml can of low sugar coffee has 8-9 grams of sugar 😨 There’s actually a lot of sugar in soft drinks that can cause your blood sugar to rise. Check the contents of your drinks to become a drink master! |

**Table S2.** Study schedule.

|  | Observation period | Intervention period | | | | Intervention sustainment period | Study drop-out or removal from study |
| --- | --- | --- | --- | --- | --- | --- | --- |
| Visit | 1 | 2 | 3 | 4 | 5 | 6 |  |
| Hospital visit day (weeks) | -2 | 0 | 4 | 8 | 12 | 24 |  |
| Hospital visit day (days) | -14 | 1 | 29 | 57 | 85 | 168 |  |
| Allotted dates (day) | -21~-7 | 1 | 22~36 | 50~64 | 78~92 | 161~175 |  |
| Consent acquisition | X |  |  |  |  |  |  |
| Inclusion and exclusion criteria evaluation | X | X |  |  |  |  |  |
| Registration and group assignment | X | X |  |  |  |  |  |
| Height | X |  |  |  |  |  |  |
| Weight, waist circumference, vitals | X | X | X | X | X | X | X |
| Physical exam | X | X | X | X | X | X | X |
| Pregnancy test (premenopausal women only) | X |  |  |  |  |  |  |
| Device use competency evaluation | X |  |  |  |  |  |  |
| Delivery of patient journals (control group only) |  | X | X | X | X |  |  |
| Collection of patient journals (control group only) |  |  | X | X | X | X | X |
| Distribution of device codes (SMC-01 group only) |  | X |  |  |  |  |  |
| Evaluation of device use condition (SMC-01 group only) |  |  | X | X | X | X | X |
| Evaluation of self-management condition (control group only) |  |  | X | X | X | X | X |
| Evaluation of past medical history and concurrent medications | X | X | X | X | X | X | X |
| Bug investigation |  | X | X | X | X | X | X |
| Adverse event investigation |  | X | X | X | X | X | X |
| Hematological examination | X | X | X | X | X | X | X |
| Blood chemistry examination | X | X | X | X | X | X | X |
| Urinalysis | X | X | X | X | X | X | X |
| HbA1c measurement | X | X | X | X | X | X | X |
| HbA1c measurement (at the hospital) | X |  |  |  |  |  |  |
| Fasting blood glucose measurement | X | X | X | X | X | X | X |
| Fasting insulin measurement |  | X | X | X | X | X | X |
| Fasting intact proinsulin measurement |  | X | X | X | X | X | X |
| C-peptide and urine albumin measurement | X |  |  |  |  |  |  |

**Table S3.** Summary of sensitivity analyses for HbA_1c_.

|  | SMC-01 group | | Control group | | Difference | | *P*-value |
| --- | --- | --- | --- | --- | --- | --- | --- |
| ANCOVA for PPS (n=63, n=51) | | | | | | | |
| week 12 | -0.10 | (-0.21 to 0.01) | 0.02 | (-0.10 to 0.15) | -0.12 | (-0.29 to 0.04) | 0.142 |
| ANCOVA for Complete Set (n=105, n=101) | | | | | | | |
| week 12 | -0.06 | (-0.15 to 0.04) | 0.05 | (-0.04 to 0.15) | -0.11 | (-0.24 to 0.03) | 0.111 |
| LMM for FAS (n=107, n=103) | | | | | | | |
| week 4 | -0.07 | (-0.15 to 0.00) | 0.00 | (-0.08 to 0.08) | -0.07 | (-0.18 to 0.04) | 0.202 |
| **week 8** | **-0.11** | **(-0.19 to -0.04)** | **0.00** | **(-0.08 to 0.08)** | **-0.12** | **(-0.23 to -0.01)** | **0.036** |
| **week 12** | **-0.05** | **(-0.13 to 0.02)** | **0.06** | **(-0.02 to 0.14)** | **-0.12** | **(-0.22 to -0.01)** | **0.038** |

Data are expressed as mean absolute change from baseline and its 95% CI.

ANCOVA, analysis of covariance; LMM, linear mixed-effects model.

Complete Set is the population for which HbA1c was measured at all time points.

Baseline measurement was measured at the start of the intervention period (Visit 2). If there was no measurement at the start of the intervention period, the last measurement before the start of the intervention period was used.

If there was no measurement at the 12-week visit, the last measurement taken between the second day and the 92nd day of the study was used in ANCOVA.

The response variable is the absolute change in HbA1c from baseline, with the baseline value as the covariate in the ANCOVA.

In the LMM, change in HbA1c was the response variable, patient was the random effect, group, time point, and group-time point interaction, and baseline HbA1c level was the fixed effect.

**Table S4.** Summary of subgroup analyses for HbA_1c_.

|  |  | SMC-01 group | | Control group | | Difference | | *P*-value |
| --- | --- | --- | --- | --- | --- | --- | --- | --- |
| Sex | | | | | | | | |
| female | week 12 | -0.14 | (-0.35 to 0.07) | 0.11 | (-0.13 to 0.34) | -0.25 | (-0.57 to 0.06) | 0.116 |
|  | week 24 | -0.02 | (-0.26 to 0.23) | 0.31 | (0.03 to 0.58) | -0.32 | (-0.69 to 0.05) | 0.086 |
| male | week 12 | -0.03 | (-0.13 to 0.08) | 0.04 | (-0.06 to 0.15) | -0.07 | (-0.22 to 0.08) | 0.336 |
|  | week 24 | 0.14 | (-0.02 to 0.31) | 0.27 | (0.1. to 0.43) | -0.12 | (-0.35 to 0.11) | 0.296 |
| Age | | | | | | | | |
| < 65 | week 12 | -0.07 | (-0.18 to 0.03) | 0.08 | (-0.03 to 0.19) | -0.16 | (-0.31 to -0.01) | 0.042 |
|  | week 24 | 0.11 | (-0.05 to 0.27) | 0.33 | (0.16 to 0.49) | -0.22 | (-0.45 to 0.01) | 0.062 |
| ≥ 65 | week 12 | 0.03 | (-0.16 to 0.22) | -0.03 | (-0.22 to 0.15) | 0.07 | (-0.20 to 0.33) | 0.624 |
|  | week 24 | 0.13 | (-0.16 to 0.41) | 0.11 | (-0.17 to 0.40) | 0.01 | (-0.39 to 0.42) | 0.950 |
| Baseline HbA1c (%) | | | | | | | | |
| < 8.0 | week 12 | -0.04 | (-0.14 to 0.06) | 0.11 | (0.01 to 0.21) | -0.15 | (-0.29 to -0.01) | 0.036 |
|  | week 24 | 0.13 | (-0.02 to 0.28) | 0.30 | (0.15 to 0.45) | -0.17 | (-0.39 to 0.04) | 0.107 |
| ≥ 8.0 | week 12 | -0.09 | (-0.28 to 0.11) | -0.08 | (-0.30 to 0.14) | 0.00 | (-0.30 to 0.29) | 0.976 |
|  | week 24 | 0.07 | (-0.23 to 0.36) | 0.22 | (-0.11 to 0.55) | -0.15 | (-0.60 to 0.29) | 0.496 |
| Baseline BMI (kg/m2) | | | | | | | | |
| < 25 | week 12 | -0.02 | (-0.17 to 0.12) | 0.07 | (-0.07 to 0.21) | -0.10 | (-0.30 to 0.10) | 0.337 |
|  | week 24 | 0.05 | (-0.14 to 0.24) | 0.28 | (0.09 to 0.46) | -0.23 | (-0.49 to 0.04) | 0.093 |
| ≥ 25 | week 12 | -0.07 | (-0.19 to 0.05) | 0.04 | (-0.09 to 0.17) | -0.11 | (-0.29 to 0.07) | 0.232 |
|  | week 24 | 0.16 | (-0.04 to 0.36) | 0.27 | (0.06 to 0.49) | -0.12 | (-0.41 to 0.18) | 0.431 |
| Hypoglycemic medication usage at baseline | | | | | | | | |
| Yes | week 12 | -0.05 | (-0.16 to 0.06) | 0.06 | (-0.04 to 0.17) | -0.11 | (-0.26 to 0.04) | 0.135 |
|  | week 24 | 0.12 | (-0.02 to 0.27) | 0.28 | (0.14 to 0.43) | -0.16 | (-0.36 to 0.05) | 0.127 |
| No | week 12 | -0.06 | (-0.25 to 0.14) | 0.01 | (-0.20 to 0.21) | -0.06 | (-0.34 to 0.22) | 0.665 |
|  | week 24 | 0.05 | (-0.39 to 0.48) | 0.23 | (-0.24 to 0.70) | -0.19 | (-0.83 to 0.45) | 0.555 |
| Self-management adherence rate | | | | | | | | |
| ≤ 80% | week 12 | 0.05 | (-0.12 to 0.21) | 0.13 | (-0.02 to 0.27) | -0.08 | (-0.30 to 0.14) | 0.458 |
|  | week 24 | 0.16 | (-0.09 to 0.41) | 0.46 | (0.23 to 0.68) | -0.30 | (-0.63 to 0.04) | 0.084 |
| > 80% | week 12 | -0.11 | (-0.22 to 0.00) | -0.01 | (-0.14 to 0.11) | -0.09 | (-0.26 to 0.07) | 0.269 |
|  | week 24 | 0.08 | (-0.08 to 0.24) | 0.10 | (-0.08 to -0.28) | -0.02 | (-0.26 to 0.22) | 0.879 |

Data are expressed as mean absolute change from baseline and its 95% CI.

Baseline measurement was measured at the start of the intervention period (Visit 2). If there was no measurement at the start of the intervention period, the last measurement before the start of the intervention period was used.

If there was no measurement at the 12-week visit, the last measurement taken between the second day and the 92nd day of the study was used.

If there was no measurement at the 24-week visit, the last measurement taken between the second day and the 175nd day of the study was used.

The response variable is the absolute change in HbA1c from baseline, with the baseline value as the covariate in the ANCOVA.

**Table S5.** All adverse events.

| System organ class | | SMC-01 group (N=107) | Control group (N=103) |
| --- | --- | --- | --- |
| Preferred term | | n (%) m | n (%) m |
|  |  |  |  |
| TEAE |  | 24 (22.4) 37 | 21 (20.4) 24 |
|  |  |  |  |
| Congenital, Familial, and Genetic Disorders | | 1 (0.9) 1 | 0 |
|  | Vitelline duct remnant | 1 (0.9) 1 | 0 |
|  |  |  |  |
| Ear and Labyrinth Disorders | | 1 (0.9) 1 | 1 (1.0) 1 |
|  | Vertigo | 1 (0.9) 1 | 0 |
|  | Tinnitus | 0 | 1 (1.0) 1 |
|  |  |  |  |
| Eye Disorders | | 0 | 2 (1.9) 2 |
|  | Subconjunctival hemorrhage | 0 | 1 (1.0) 1 |
|  | Dry eye | 0 | 1 (1.0) 1 |
|  |  |  |  |
| Gastrointestinal Disorders | | 4 (3.7) 5 | 3 (2.9) 3 |
|  | Cheilitis | 1 (0.9) 1 | 0 |
|  | Cavity | 1 (0.9) 1 | 1 ( 0.9) 1 |
|  | Gastric ulcer | 1 (0.9) 1 | 0 |
|  | Melena | 1 (0.9) 1 | 0 |
|  | Loose stools | 1 (0.9) 1 | 0 |
|  | Diarrhea | 0 | 1 (1.0) 1 |
|  | Gastroesophageal Reflux Disease | 0 | 1 (1.0) 1 |
|  |  |  |  |
| General Disorders and Administration Site Conditions | | 1 (0.9) 1 | 0 |
|  | Pain | 1 (0.9) 1 | 0 |
|  |  |  |  |
| Infectious Diseases and Parasitic Infections | | 5 (4.7) 5 | 6 (5.8) 7 |
|  | Appendicitis | 1 (0.9) 1 | 0 |
|  | Epipharyngitis | 1 (0.9) 1 | 2 (1.9) 2 |
|  | Periodontitis | 1 (0.9) 1 | 1(1.0) 1 |
|  | Pyelonephritis | 1 (0.9) 1 | 0 |
|  | Sinusitis | 1 (0.9) 1 | 0 |
|  | Shingles | 0 | 1 (1.0) 1 |
|  | Tonsillitis | 0 | 1 (1.0) 1 |
|  | Oral herpes | 0 | 1 (1.0) 1 |
|  | COVID-19 | 0 | 1 (1.0) 1 |
|  |  |  |  |
| Injury, Poisoning, and Procedural Complications | | 3 (2.8) 4 | 1 (1.0) 1 |
|  | Humerus fracture | 1 (0.9) 1 | 0 |
|  | Contusion | 1 (0.9) 1 | 0 |
|  | Tooth fracture | 1 (0.9) 2 | 0 |
|  | Vertebral compression fracture | 0 | 1 (1.0) 1 |
|  |  |  |  |
| Clinical Tests | | 2 (1.9) 2 | 2 (1.9) 2 |
|  | Elevated alanine aminotransferase | 1 (0.9) 1 | 0 |
|  | Urinary occult blood positive | 1 (0.9) 1 | 0 |
|  | Elevated blood triglycerides | 0 | 1 ( 1.0) 1 |
|  | Elevated platelet count | 0 | 1 ( 1.0) 1 |
|  |  |  |  |
| Musculoskeletal and Connective Tissue Disorders | | 6 (5.6) 6 | 2 (1.9) 2 |
|  | Back Pain | 3 (2.8) 3 | 1 (1.0) 1 |
|  | Arthritis | 1 (0.9) 1 | 0 |
|  | Myalgia | 1 (0.9) 1 | 0 |
|  | Spinal disc herniation | 1 (0.9) 1 | 0 |
|  | Periarthritis | 0 | 1 (1.0) 1 |
|  |  |  |  |
| Nervous System Disorders | | 1 (0.9) 1 | 0 |
|  | Hypoesthesia | 1 (0.9) 1 | 0 |
|  |  |  |  |
| Psychiatric Disorders | | 0 | 1( 1.0) 1 |
|  | Sleep Disorders | 0 | 1 (1.0) 1 |
|  |  |  |  |
| Renal and Urinary Disorders | | 1 (0.9) 1 | 0 |
|  | Hematuria | 1 (0.9) 1 | 0 |
|  |  |  |  |
| Reproductive System and Breast Disorders | | 1 (0.9) 1 | 0 |
|  | Benign prostatic hyperplasia | 1 (0.9) 1 | 0 |
|  |  |  |  |
| Respiratory, Thoracic, and Mediastinal Disorders | | 1 (0.9) 1 | 1(1.0) 1 |
|  | Dyspnea | 1 (0.9) 1 | 0 |
|  | Pharyngeal edema | 0 | 1 (1.0) 1 |
|  |  |  |  |
| Skin and Subcutaneous Tissue Disorders | | 6 (5.6) 7 | 3 (2.9) 3 |
|  | Contact Dermatitis | 3 (2.8) 3 | 0 |
|  | Dyshidrotic Eczema | 1 (0.9) 1 | 0 |
|  | Eczema | 1 (0.9) 1 | 0 |
|  | Urticaria | 1 (0.9) 1 | 1 (1.0) 1 |
|  | Asteatosis cutis | 1 (0.9) 1 | 0 |
|  | Dry Skin | 0 | 1 (1.0) 1 |
|  | Rash | 0 | 1 (1.0) 1 |
|  |  |  |  |
| Vascular Disorders | | 1 (0.9) 1 | 1 (1.0) 1 |
|  | Hypertension | 1 (0.9) 1 | 1 (1.0) 1 |

MedDRA/J Version 23.0

TEAE = Treatment-emergent Adverse Event.

TEAEs are adverse events/malfunctions that occurred from the start of intervention period to the final measurement/time of drop-out.

Percentages were calculated based on the total number of subjects in the analysis group for each assigned treatment group.

The sort order is System Organ Class (SOC) in SOC code order, Preferred Term (PT) in descending order of frequency in the SMC-01 group, and PT code order.

The number of occurrences was counted as one case if the same SOC/PT event was reported in the same subject.

n = number of occurrences, m = number of events.

**Table S6.** Breakdown of bugs.

|  | SMC-01 group (n = 107) |
| --- | --- |
|  | n (%) m |
| Bug | 46 (43.0) 85 |
| Malfunction or freeze | 39 (36.4) 61 |
| Network connectivity issue | 1 (0.9) 1 |
| Incomplete data synchronization | 17 (15.9) 23 |

The number of occurrences was counted as one case if the same bug was reported in the same subject. n = number of occurrences, m = number of events.

**Section S2**. PPS definition. PPS: per protocol set.

The PPS included participants meeting all the following criteria:

- Met the inclusion criteria and did not meet the exclusion criteria
- HbA1c value both at baseline and at the end of the intervention period is not missing
- No use of any of the prohibited medications, used conditional medications according to the study guidelines, and did not carry out any other prohibited treatments
- Self-management adherence rate above 80%

Did not commit any serious study protocol violations by the end of the intervention period

**Section S3.** Safety analysis.

The frequency of adverse events (Table S5) was 22.4% (24/107 participants, 37 events) in the SMC-01 group and 20.4% (21/103 participants, 24 events) in the control group, with no statistically significant difference between the two groups (*P* = 0.739). Adverse events or deaths caused by the device under study did not occur in either group. Four critical adverse events occurred in 3.7% (4/107 participants) of the SMC-01 group participants and no critical adverse events occurred in the control group participants (*P* = 0.122). Critical adverse events in the SMC-01 group included appendicitis, pyelonephritis, humerus fracture, and bruise which each occurred once. Discontinuation of the trial therapy due to an adverse event did not occur in either group of the study.

43.0% (46/107 patients) of the SMC-01 group encountered bugs (Table S6), none of which were related to adverse events. Most bugs resolved upon software update.
